# TNF induces glycolytic shift in fibroblast like synoviocytes via GLUT1 and HIF1A

**DOI:** 10.1038/s41598-021-98651-z

**Published:** 2021-09-29

**Authors:** Kathrin Koedderitzsch, Ekaterina Zezina, Lingzi Li, Matthias Herrmann, Nadine Biesemann

**Affiliations:** 1 Sanofi Immunology and Inflammation Research Therapeutic Area, Type 1/17 Immunology Cluster, Industriepark Hoechst, 65926 Frankfurt am Main, Germany; 2 Present Address: BioNTech, An der Goldgrube 12, 55131 Mainz, Germany

**Keywords:** Autoimmunity, Cytokines, Translational immunology, Mechanisms of disease, Rheumatoid arthritis

## Abstract

TNF is a central cytokine in the pathogenesis of rheumatoid arthritis (RA). Elevated level of TNF causes local inflammation that affects immune cells and fibroblast-like synoviocytes (FLS). Nowadays, only 20–30% of patients experience remission after the standard of care therapy—antibodies against TNF. Interestingly, responders show reduced levels of GLUT1 and GAPDH, highlighting a potential link to cellular metabolism. The aim of the study was to investigate whether TNF directly affects the metabolic phenotype of FLS. Real-time respirometry displayed TNF-induced upregulation of glycolysis along with a modest increase of oxidative phosphorylation in FLS from healthy donors. In addition, TNF stimulation enhanced HIF1A and GLUT1 expression. The upregulation of HIF1A and GLUT1 reflects their enriched level in FLS from RA patients (RA-FLS). The inhibition of TAK1, HIF1a and hexokinase deciphered the importance of TNF/TAK1/HIF1A/glycolysis signaling axis. To prove that inhibition of glycolysis reduced the pathogenic phenotype, we showed that 2-deoxyglucose, a hexokinase inhibitor, partially decreased secretion of RA biomarkers. In summary, we identified a direct role of TNF on glycolytic reprogramming of FLS and confirmed the potency of immunometabolism for RA. Further studies are needed to evaluate the therapeutic impact especially regarding non-responder data.

## Introduction

Inflamed synovial cavity in RA is characterized by infiltration of immune cells and stromal cell activation^1,2^. Fibroblast-like synoviocytes (FLS) together with macrophage-like synovial cells form the intimal lining layer of the synovial membrane. FLS proliferation and accumulation of immune cells trigger the inflammation and lead to membrane thickness, known as synovial hyperplasia – one of the primary symptoms of RA. Along with immune cells, FLS are considered the key players in the development of RA^3^. FLS from RA patients (RA-FLS) share several characteristics with tumor cells e.g. increased proliferation rate and apoptosis resistance^4–7^. The microenvironment of inflamed cartilage comprises a mixture of cytokines, that are secreted by FLS and immune cells. TNF promotes the generation of cytokines and chemokines, expression of endothelial cell adhesion molecules, angiogenesis and induction of pain in RA^8^. TNF mediates cell proliferation and survival via nuclear factor ´kappa-light-chain-enhancer´ of activated B-cells (NF-kB) and mitogen-activated protein kinases (MAPK) pathways^9^. One of the essential steps in NF-kB activation is receptor-interacting protein (RIP) mediated TAK1 (TGF-Beta Activated Kinase 1) recruitment^10^. The relevance of TNF in several autoimmune diseases is well-known and illustrated by five approved antibodies. Despite reducing synovial inflammation, the current standard of care therapy do not cure the disease and only 20–30% of patients experience remission^11^.

Our interest is focused on the fact that patients responding to anti-TNF therapies showed a downregulation of GLUT1 and GAPDH, a rate-limiting enzyme in glycolysis^12^. Furthermore, a decreased glucose uptake was demonstrated using FDG-PET/CT in patients after anti-TNF treatment^13,14^. In general upregulated glucose metabolism in RA-FLS was already published in 1991 as a specific feature of RA disease development^15^. In addition, RA-FLS are more glycolytic than FLS from OA patients^16^. Nutrient demand is much higher in RA-FLS to enable rapid growth and cytokine production^17^. The significant upregulation of GLUT1 and reduction in mitochondrial respiration confirms the importance of metabolic changes in RA-FLS^16,18,19^.

We questioned whether a single cytokine like TNF is able to induce metabolic reprogramming and pro-inflammatory phenotype in FLS from healthy donors (H-FLS) similarly to the described phenotype of RA-FLS.

## Methods

### Material

Fibroblast-like synoviocytes from healthy and RA donors and the corresponding Synoviocyte Growth Medium were commercially purchased from Cell Applications, Inc. Glucose, oligomycin, HIF inhibitor V and 2-Deoxyglucose were from Sigma-Aldrich. Recombinant human TNF was from R&D systems, (5Z)-7-oxozeanol from Tocris. We used a highly potent Glut1 inhibitor from the 1H-pyrazolo[3,4-d]pyrimidine class.

### Seahorse

20.000 H-FLS were seeded in a Seahorse 96 well-plate (XF96 cell culture microplate). Cells were stimulated on the following day with recombinant TNF with and without inhibitors for 24 h. On the day of the experiment, first the medium was removed from the cell plate and the cells were washed three times with Seahorse XF base medium with 3 mM l-glutamine and incubated for 45 min at 37 °C. Afterwards glycolysis stress test was performed in the Seahorse XFe 96 analyzer (Seahorse Bioscience) in real-time according to the protocol provided by the manufacturer. Glycolysis, Glycolytic capacity, and glycolytic reserve were studied by treatment of FLS subsequently with 24 mM glucose, 1 µM Oligomycin and 100 mM 2-deoxyglucose. Measurements were done in at least 6 replicates per plate and normalized to the amount of protein using bicinchoninic acid (BCA) assay (Pierce® BCA Protein Assay Kit, ThermoFisher Scientific).

### Luminex

FLS were stimulated after overnight attachment to 96 well-plates with recombinant TNF with and without inhibitors for 24 h. Afterwards supernatants were collected and frozen at − 20 °C. The analysis was carried out according to the manufacturer´s user guide (ProcartaPlex™Multiplex Immunoassay, Invitrogen ThermoFisher scientific) using the ProcartaPlex™Multiplex Immunoassay Kit.

### RT-PCR TaqMan

RNA was isolated from stimulated or unstimulated FLS with the RNeasy® Mini Kit from QIAGEN. The isolation was carried out according to the manufacturer protocol (RNeasy® Mini Kit, Quick-Start Protocol, QIAGEN) using the automated system QIAcube. After reverse transcription with the Applied Biosystems ™ High Capacity cDNA Reverse Transcription Kit from Thermo Fisher, TaqMan Assay was performed with the ViiA™7 Real Time PCR System from Applied Biosystems®. For quantitative normalization (endogenous control) the housekeeping gene RPL37a was used. For the relative quantification of the obtained results the comparative CT method was chosen.

### Comparison of gene expression from multiple transcriptomics studies in RA

Expression of certain genes was queried in archival studies comparing RA case and control synovial tissues. Records for this search were extracted from OmicSoft DiseaseLand Database, release HumanDisease_B37 (OmicSoft Corporation, 2015). Analyzed datasets series: GSE89408^20^, GSE77298^21^, GSE29746^22^, GSE1919^23^. Heatmap comparison of these search results was done with R package pheatmap^24^.

### Statistical evaluation

The statistical analysis of the experiments was done using Microsoft Excel 2010 (Microsoft Corporation) and GraphPad Prism 7.02 (GraphPad Software). To calculate p-values, the unpaired t-test for the comparison of two groups or the one-way analysis of variance (ANOVA) followed by pairwise comparison were used. Visualization was done using RStudio (Version 1.3.959, with R version 4.0.2). R package tidyverse^25^ was used, and pheatmap^24^ was applied with agglomerative clustering method (Euclidean distance) for hierarchical clustering.

## Results

### TNF induces metabolic shift in H-FLS

**Figure 1:**
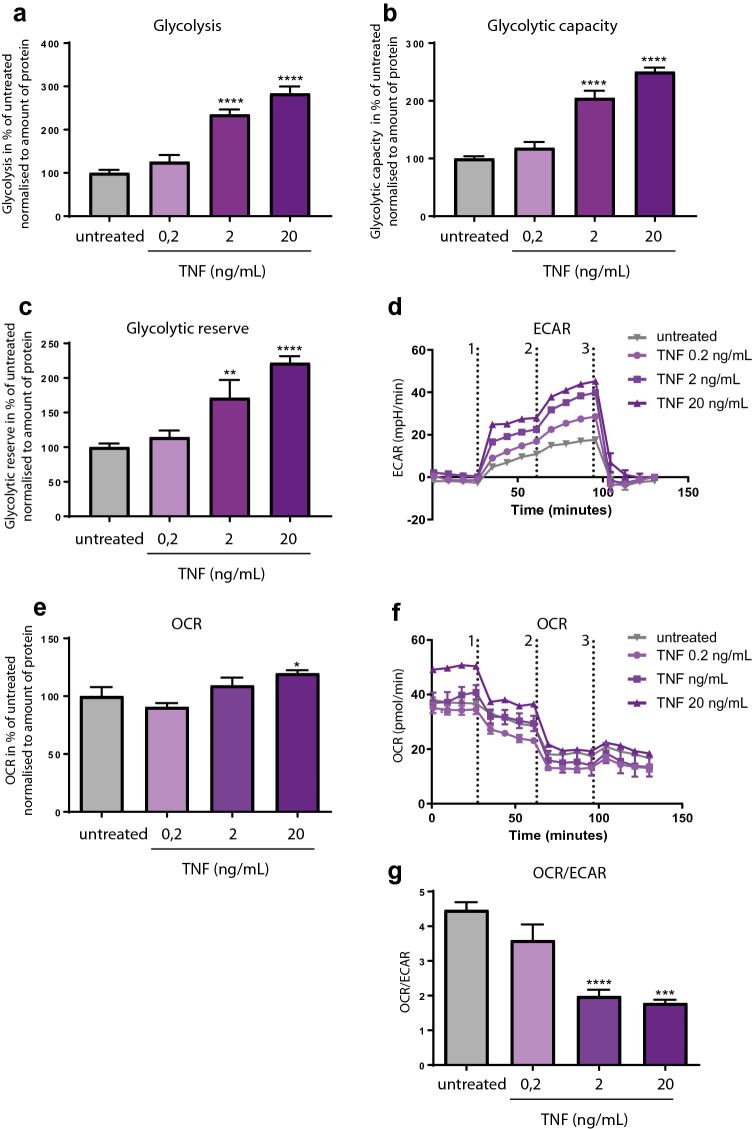
TNF activates glycolysis and mitochondrial respiration in H-FLS. Real-time analysis of glycolytic function in FLS from three healthy donors after 24 h incubation with recombinant human TNF using Seahorse XFe96. Glycolysis stress test with injection of glucose (24 mM), oligomycin (1 µM) and 2-DG (100 mM). (**a**) Glycolysis, (**b**) Glycolytic capacity, (**c**) Glycolytic reserve, (**d**) Representative Seahorse ECAR graph from one experiment. 1—injection of 24 mM glucose, 2—1 µM Oligomycin and 3—100 mM 2-deoxyglucose. (**e**) OCR, (**f**) Representative Seahorse OCR graph from one experiment. 1—injection of 24 mM glucose, 2—1 µM Oligomycin and 3—100 mM 2-deoxyglucose. (**g**) OCR/ECAR after glucose injection (experiments performed with 3–6 technical replicates, 2 independent experiments, 3 different donors). Data were normalized to protein content using BCA assay. Values represent means ± SEM. *P ≤ 0.05; *P ≤ 0.01; ***P˂0.001; ****P˂0.0001, unpaired one-way Anova with Dunnett correction.

TNF is a pleiotropic cytokine that activates diverse pathways of apoptosis and inflammation. One of the anti-TNF treatment outcomes in responders is a reduction of glucose uptake^26^. To find out whether stimulation with TNF directly promotes metabolic changes in FLS (or it is rather a consequence of inflammation induction), Seahorse glycolysis stress test was performed in FLS from healthy donors (H-FLS) upon treatment with TNF. Thereby we can analyze real-time consumption of glucose as well as the glycolytic capacity and glycolytic reserve of H-FLS by injection of different substrates using extracellular acidification rate (ECAR) as parameter for proton release by glycolysis. Our Seahorse data indicate that TNF stimulation dose dependently increases glycolysis, glycolytic capacity, and glycolytic reserve (Fig. 1a–d). The other parameter, evaluated by Seahorse, is called oxygen consumption rate (OCR). OCR reports mitochondrial respiration and therefore it allows to estimate mitochondrial activity and their contribution to energy production. In contrast to ECAR, oxygen consumption is only slightly increased at the highest TNF concentration (Fig. 1e,f). These data are confirmed by the OCR/ECAR ratio highlighting TNF induced metabolic reprogramming in FLS towards a more glycolytic phenotype (Fig. 1g). Next, we aimed to understand whether TNF would also induce glycolysis in FLS from RA patients. Repeating this experiment with RA-FLS we observed a comparable increase in glycolysis with the highest TNF concentration (Suppl. Fig. S1).

### TNF induced metabolic shift in H-FLS reflects RA-FLS metabolic features

**Figure 2:**
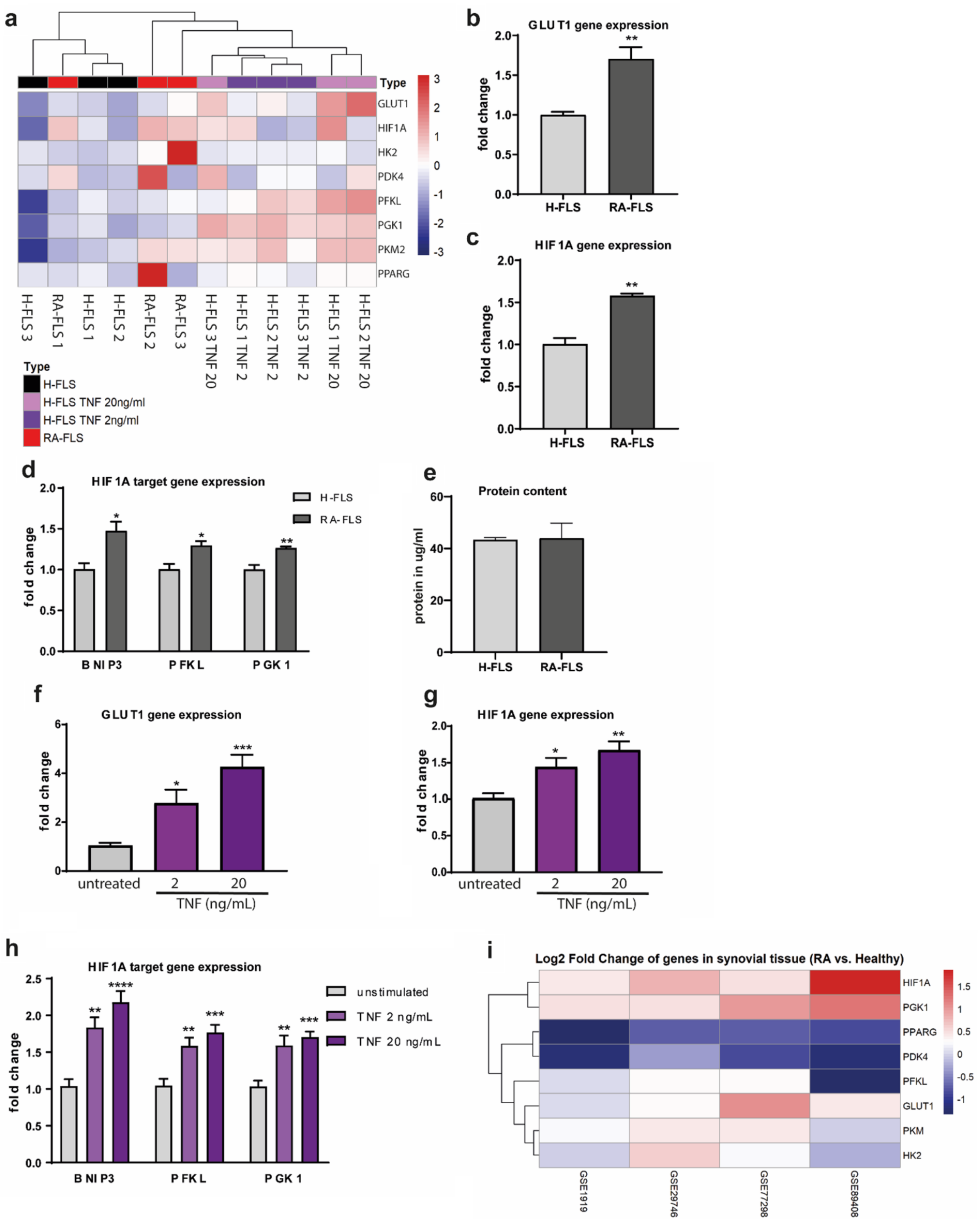
Upregulation of GLUT1 and HIF1A signaling in H-FLS after TNF stimulation and in RA-FLS. Metabolic gene expression in H-FLS and RA-FLS, and H-FLS after 24 h stimulation with recombinant human TNF (2 ng/mL, 20 ng/mL, 3 donors each). (**a**) Hierarchical agglomerative clustering with Euclidean distance, fold change. (**b**–**d**) Expression of GLUT1 (**b**), HIF1A (**c**) and HIF1A target genes (**d**) in fibroblast-like synoviocytes from healthy or RA donors. (**e**) Protein content was analyzed using BCA assay. (**f**,**g**) Metabolic gene expression in H-FLS after stimulation with recombinant TNF. Fibroblast-like synoviocytes from three healthy donors were stimulated with recombinant human TNF (2 and 20 ng/ml) for 24 h and expression of GLUT1 (**f**), HIF1A (**g**) and HIF1A target genes (**h**) was evaluated (2 independent experiments, 3 different donors, n = 6). (**i**) Heatmap of DiseaseLand analysis from 4 independent studies, scale reflects fold changes. Values represent means. ± SEM, unpaired t-test (**b**–**e**) or unpaired one-way Anova with Dunnett correction (**f**,**g**).

To prove that stimulation of fibroblast-like synoviocytes from healthy donors with TNF resembles the metabolic phenotype of FLS in RA joint, the expression level of crucial metabolic genes was compared in H-FLS after treatment with TNF versus RA-FLS. Hierarchical clustering of several metabolic targets highlighted that TNF stimulated H-FLS from different donors cluster together and are more similar to RA-FLS compared to unstimulated H-FLS (Fig. 2a). Results needs to be taken with caution as only two out of three RA donors cluster together. The expression level of GLUT1 (Fig. 2b), HIF1A (Fig. 2c), phosphofructokinase (PFKL), and phosphoglycerate kinase (PGK1) were upregulated in RA-FLS (Fig. 2d) without any difference on protein content (Fig. 2e). Interestingly, TNF promotes the expression of the same genes in H-FLS (Fig. 2f–h). The above-mentioned genes are involved in glycolysis and its regulation. Further analysis of the data revealed that expression level of hexokinase 2 (HK2), pyruvate dehydrogenase kinase 4 (PDK4), pyruvate kinase muscle isozyme M2 (PKM2), peroxisome proliferator-activated receptor gamma (PPARG) and PFKFB3 were—despite a trend—not significantly different between H-FLS and RA-FLS (Fig. 2a, Suppl. Fig. S2a). Similarly, the expression of these gene tended to be enhanced by TNF treatment in H-FLS and RA-FLS (Fig. 2a, Suppl. Figs. S1f, S2b). The main difference between the tested metabolic genes was seen for PKM2, which was induced by TNF (Fig. 2a, Suppl. Fig. S2b), but not upregulated in RA-FLS (Suppl. Fig. S2a). To make a comprehensive conclusion on the relevance of HK2, PDK4, PKM2, and PPARG for RA pathogenesis more samples need to be analyzed. Therefore, we analyzed four publicly available datasets comparing synovial tissue of RA patients and healthy donors. We confirmed induction of HIF1A and PGK1 in all four datasets (Fig. 2i) and observed induction of GLUT1 (SLC2A1), HK2, PFKL and PKM2 in some studies. The studies differed in several aspects: technology (first three studies are MicroArray, GSE89408 is RNAseq), patient number (biggest study was GSE89408), disease stage and duration, biopsy material and treatments. Unfortunately, we do not have the patient information to focus our analysis on anti-TNF treated nor can’t we exclude those.

### TNF signaling through TAK1 activates glycolysis

**Figure 3:**
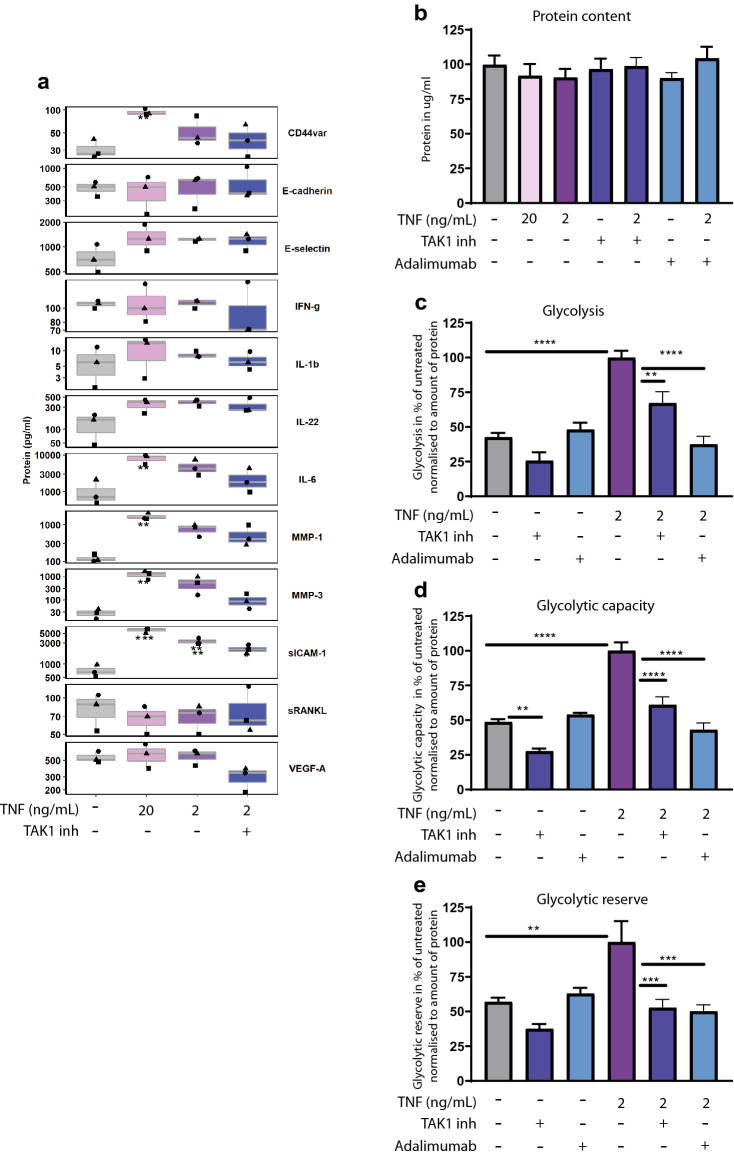
TNF induces glycolytic reprogramming via TAK1. H-FLS from three donors were stimulated with recombinant human TNF, TAK1 inhibitor (5Z)-7-Oxozeaenol (0.5 µM) or the anti-TNF antibody Adalimumab (50 nM) at indicated concentrations for 24 h. (**a**) Luminex® Multiplex assay to evaluate cytokines and disease-relevant biomarkers in cell culture supernatants (3 donors, 1 experiment). One dot represents one donor, donors can be differentiated based on the shape. Significance to unstimulated are highlighted. (**b**) Protein content was analyzed using BCA assay 24 h after stimulation (experiments performed with 6 technical replicates, 1–2 independent experiments, 3 different donors). (**c**–**e**) Real-time analysis of the glycolytic function of FLS using Seahorse XFe96. Glycolysis stress test with injection of glucose (24 mM), oligomycin (1 µM) and 2-DG (100 mM). (**c**) Glycolysis (**d**) Glycolytic capacity (**e**) Glycolytic reserve. Data were normalized to protein content using BCA assay (experiments performed with 6 technical replicates, 1–2 independent experiments, 3 different donors). Values represent means ± SEM (b-e) or are depicted as boxplot with median and first and third quartile (a). *P ≤ 0.05; **P˂0.01; ***P˂0.001; ****P ≤ 0.0001, one-way ANOVA with Tukey multiple pairwise-comparisons test (a), unpaired one-way Anova with Dunnett correction (**b**–**e**).

**Figure 4:**
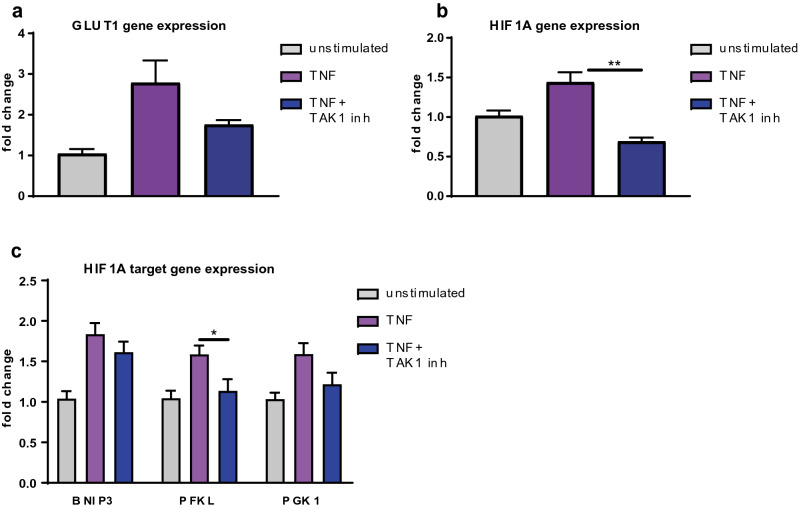
TNF induces upregulation of GLUT1 and HIF1A via TAK1. (**a**–**c**) Fibroblast-like synoviocytes from three healthy donors were stimulated with recombinant human TNF (2 ng/mL) and the TAK1 inhibitor (5Z)-7-oxozeaenol (0.5 µM) for 24 h and expression of GLUT1 (**a**), HIF1A (**b**) and HIF1A target genes (**c**) was evaluated (n ≥ 3, 3 different donors). Values represent means ± SEM. *P ≤ 0.05; **P˂0.01. Unpaired one-way Anova with Dunnett correction.

We verified a similar glycolytic signature in RA-FLS and in healthy FLS after TNF stimulation. Next, we wondered how TNF mediates the induction of GLUT1 and HIF1A. TNF is a pleiotropic cytokine regulating cell proliferation and inflammatory responses via NF-kB and MAP kinase pathways. To prove the mode of action we stimulated H-FLS with TNF and analyzed several cytokines and biomarkers such as matrix metalloproteases (MMP). TNF dose-dependently upregulated known downstream targets like IL-6 (Fig. 3a). In addition, TNF stimulated secretion of disease biomarkers involved in the pathogenesis of RA like MMP-1, MMP-3 and Intercellular Adhesion Molecule 1 (ICAM1) (Fig. 3a). To decipher the downstream pathway promoting metabolic changes and secretion of RA biomarkers, NF-κB activation was blocked by 5Z-7-Oxozeaenol, a TAK1 inhibitor. The chemical inhibition of TAK1 tended to reduce IL-6 and MMP-3 level after TNF stimulation (Fig. 3a). Neither the TAK1 inhibitor nor TNF itself influenced protein content (Fig. 3b). Moreover, TAK1 inhibition reverted TNF-induced glycolysis, glycolytic capacity, and glycolytic reserve in H-FLS (Fig. 3c–e) pointing to the connection between inflammation and metabolic changes. The effect of TAK1 inhibition on metabolic reprogramming was comparable to the positive control—an approved anti-TNF antibody Adalimumab (Fig. 3c–e) highlighting that metabolic effects of TNF are mediated via TAK1. Interestingly 5Z-7-Oxozeaenol reduced the glycolytic reserve already in unstimulated cells (Fig. 3c) implying a link between TAK1 and glycolytic flexibility. In addition to direct effects on the functional glycolysis parameters, inhibition of TAK1 also showed a trend towards reduced TNF-induced GLUT1 induction (Fig. 4a). These data are supported by significant inhibition of HIF1A and PFKL expression (Fig. 4b,c) confirming the link between TNF/TAK1 signaling and metabolic changes.

### Secretion of TNF-induced biomarkers is partially dependent on glycolysis

**Figure 5:**
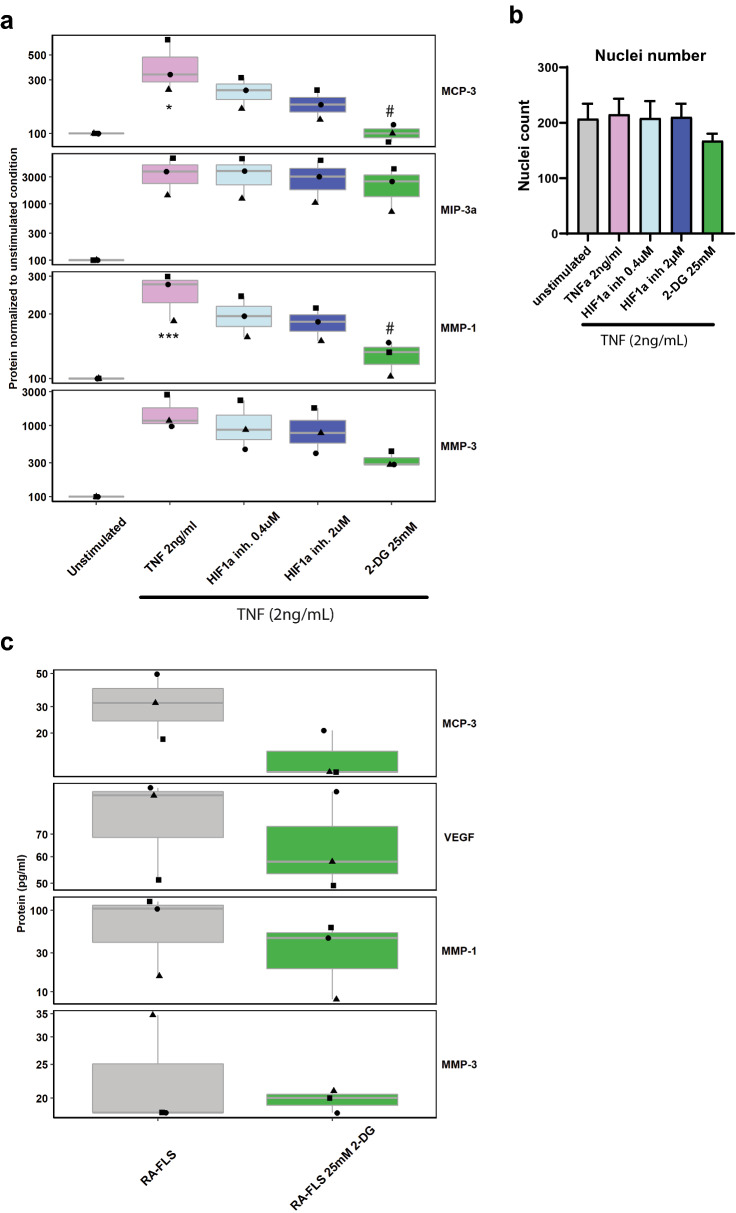
Inhibition of glycolysis reduces biomarker secretion in FLS. (**a**-**b**) Fibroblast-like synoviocytes from three healthy donors were stimulated with recombinant human TNF (2 ng/ml) and the HIF1A inhibitor HIF V or 2-deoxyglucose, a competitive inhibitor of hexokinase for 24 h in the indicated concentrations. (**a**) Luminex®Multiplex assay to evaluate cytokines and disease-relevant biomarkers in cell culture supernatants (n = 3 different donors, experiments were performed in 3 technical replicates). One-way ANOVA with Tukey multiple pairwise-comparisons. Data are shown as boxplot with median and first and third quartile. Donors are highlighted with different shapes. ***P˂0.001 vs. unstimulated control, #P<0.05 vs. TNF stimulation. (**b**) Nuclei number (**c**) Fibroblast-like synoviocytes from three RA patients were treated with 24 mM 2-deoxyglucose (2-DG), a competitive inhibitor of hexokinase (24 h). Luminex®Multiplex assay to evaluate cytokines and disease-relevant biomarkers in cell culture supernatants (n = 3 different donors, experiments were performed in 3 technical replicates). One-way ANOVA with Tukey multiple pairwise-comparisons. Data are shown as boxplot with median and first and third quartile. Donors are highlighted with different shapes.

We observed TNF-induced glycolytic reprogramming of healthy FLS and a similar glycolytic signature in FLS from RA donors. Next, we wondered whether inhibition of HIF1A and glycolysis would block TNF-induced proinflammatory effects in healthy FLS similar to TAK1 inhibition. We focused on the major TNF-dependent RA biomarkers described before (Fig. 3a) e.g. pro-inflammatory chemokines and matrix metalloproteases. Inhibition of HIF1A showed a trend towards partial reduction in these biomarkers’ secretion, although effects were not significant (Fig. 5a). It should be mentioned that HIF1A inhibitors act via different mode of actions which might be responsible for the partial effects observed. Broad blockade of glycolysis by inhibition of the rate-limiting enzyme hexokinase with 2-deoxyglucose (2-DG) significantly reduced the secretion of MMP1 and MCP-3 with tendencies on MMP-3 (Fig. 5a). Nuclei number as marker of potential cell death was not or only modestly impacted (Fig. 5b) highlighting that the effects on biomarkers were not induced by toxicity of the compounds used. Of note, similar experiments performed with an inhibitor of GLUT1 did not affect biomarker secretion (Suppl. Fig. S3a). Similarly, GLUT1 inhibition did not affect TNF-induced HIF1A upregulation (Suppl. Fig. S3b) arguing for compensatory mechanisms by other glucose transporters.

### Secretion of RA biomarkers is partially dependent on glycolysis in RA-FLS

We compared whether the above mentioned biomarkers are upregulated in RA-FLS. We observed—despite a high donor variation—a trend towards increased secretion of these biomarkers in RA-FLS compared to healthy controls (Suppl. Fig. S4). Similar to the experiments with TNF stimulation in H-FLS, glycolysis inhibition with 2-DG tended to reduce secretion of MCP-3 and MMP1 in FLS from RA patients (Fig. 5c). Due to the high donor variation, the effect did not reach statistical significance.

## Discussion

The first evidence of glycolysis upregulation in RA joints was elevated lactate and reduced glucose levels in synovial fluid^27^. Additionally, MRI and PET results confirmed increased uptake of labelled fluorodeoxyglucose in RA patients^28^. A rat arthritis model suggested that cells from synovial pannus contribute to enhanced glucose consumption rather than infiltrated immune cells^29^. In addition, hexokinase 2 (HK2), a rate-limiting enzyme of glycolysis, is known to regulate FLS aggressive functions^30^. Our data show high expression level of GLUT1, HIF1A, and its glycolytic downstream targets in RA-FLS compared to H-FLS. These results support already published data describing changes between FLS from osteoarthritis (OA) and RA patients^16^. Enhanced glucose uptake, glycolytic rate, as well as increased levels of GLUT and HIF1A are known for RA-FLS^16–18^.

In our study treatment with TNF induces glycolysis and expression of metabolic genes in H-FLS. TNF is a crucial cytokine that mediates RA pathology. Anti-TNF therapy diminishes inflammation and downregulates glucose uptake in RA joints^13,26^. Moreover, responders to TNF blockade display lower level of GLUT1 and GAPDH expression^12^. Very few papers are focused on the impact of TNF on FLS. We demonstrate that TNF promotes secretion of RA biomarkers e.g. pro-inflammatory cytokines and MMPs in H-FLS. Furthermore, TNF induces glycolysis and expression of metabolic genes. Recently the link between TNF signaling and glycolysis was suggested in different cell types. In endothelial cells TNF increases 6-phosphofructo-2-kinase/fructose-2,6-biphosphatase 3 (PFKFB3) expression and inhibition of PFKFB3 reduces TNF-induced monocyte adhesion and transmigration^31^. We also observed a slight increase in PFKFB3 after TNF stimulation in healthy FLS supporting a general role for TNF in glycolysis. According to published data the exposure to TNF enhances the NF-κB pathway in FLS^32^ and alters chromatin states^33^. TAK1 binding to TNF receptor 1 is shown to be essential for TNF-induced NF-κB activation^10^. We show that TAK1 inhibition attenuates TNF-induced secretion of RA biomarkers and metabolic changes in H-FLS.

Interestingly, Biniecka et al. hypothesized a connection between TNF, glycolysis, and hypoxia. The study determines an inverse correlation between tissue oxygen levels, glycolytic gene expression and lactate levels in FLS cultures as well as in synovial biopsies from anti-TNF responders vs. anti-TNF non-responders^12^. Hypoxia induced accumulation of HIF1A modulates the expression and distribution of GLUT1 in many cell types^34–36^**.** HIF1A activation promotes glycolysis, ROS production and epithelial–mesenchymal transition in RA-FLS in vitro^37^. Thus, HIF1A induced GLUT1 upregulation is a potential mechanism of RA-FLS phenotype development. Our analysis of the potential impact of HIF1A and GLUT1 inhibition on cytokine secretion was probably limited by the availability of potent HIF1A inhibitors, their variable mode of actions and compensatory mechanisms from glucose transporters. The used HIF inhibitor promotes for example HIF1A degradation via upregulation of von Hippel-Lindau expression in hypoxia. We therefore focused on the importance of glycolysis by using 2-DG, a competitive inhibitor of glucose. Inhibition of glycolysis with 2-DG partly blocked TNF’s proinflammatory effects on MMP1 and MCP-3 highlighting the strong connection between cytokine signaling and cellular metabolism. Moreover, 2-DG treatment inhibited RA biomarkers secretion, supporting the idea of a therapeutic potential for glycolysis inhibition in RA-FLS, innate and adaptive immune cells and in vivo disease models^38^. To our knowledge our work is the first one displaying glycolysis inhibition in unstimulated RA-FLS with anti-inflammatory effects.

**Figure 6:**
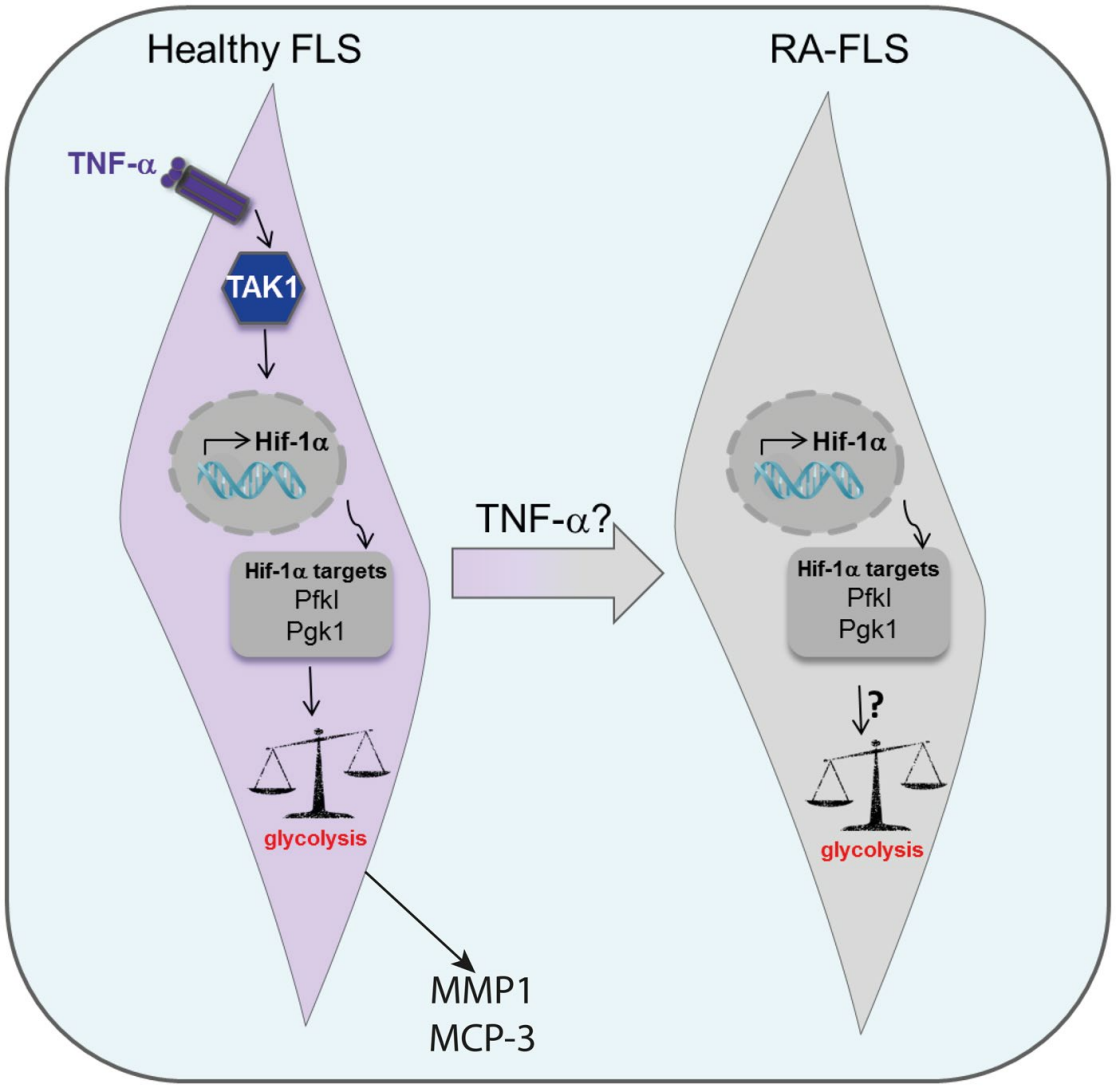
Hypothesis. TNF induces HIF1A and glycolytic gene expression via TAK1 in fibroblast-like synoviocytes from healthy donors, which is reflected in increased glycolysis analysed by Seahorse measurements. Inhibition of glycolysis reduces TNF-induced upregulation of RA biomarker like MMP1 and MCP-3. Similarly, FLS from RA patients showed an upregulation of these genes, leading to the hypothesis that TNF might be one of the cytokines driving metabolic reprogramming in Rheumatoid Arthritis joints.

Finally, we identified a direct impact of TNF on H-FLS (and RA-FLS) metabolism. TNF stimulation seems to reflect the metabolic and pro-inflammatory phenotype of RA-FLS via HIF1A and GLUT1 signaling (Fig. 6). These results correlate with previously published anti-TNF responder data and highlight the potential of GLUT1 and glucose metabolism as target engagement and disease response biomarker in the clinic. Further studies are needed to evaluate whether this metabolic signature could be used for patient stratification and whether these effects can be detected in human blood as well.

## Supplementary Information

Supplementary Information.

## Data Availability

The datasets used and/or analyzed during the current study are included in this article.

## Abbreviations

2-DG: 2-Deoxyglucose
FDG-PET: Fluordesoxyglucose positron emission tomography
FLS: Fibroblast-like synoviocytes
GAPDH: Glyceraldehyde 3-phosphate dehydrogenase
GLUT1: Glucose transporter 1
H-FLS: Fibroblast-like synoviocytes from healthy donor
HIF1A: Hypoxia-Inducible Factor-1
HK2: Hexokinase 2
ICAM1: Intercellular Adhesion Molecule 1
MAPK: Mitogen-activated protein kinase
MMP: Matrix metalloprotease
NF-kB: Nuclear factor ‘kappa-light-chain-enhancer’ of activated B-cells
OA: Osteoarthritis
PDK4: Pyruvate dehydrogenase kinase 4
PFKFB3: 6-Phosphofructo-2-kinase/fructose-2,6-bisphosphatase-3
PFKL: Phosphofructokinase
PGK1: Phosphoglycerate kinase
PK: Pyruvate kinase
PPARG: Peroxisome proliferator-activated receptor gamma
RA: Rheumatoid arthritis
RA-FLS: Fibroblast-like synoviocytes from RA donor
RIP: Receptor-interacting protein
TAK1: TGF-Beta Activated Kinase 1
TNF: Tumor Necrosis Factor alpha
