# Supplementary material for: TNF induces glycolytic shift in fibroblast like synoviocytes via GLUT1 and HIF1A
Source: Sci Rep. 2021 Sep 29;11:19385. doi: 10.1038/s41598-021-98651-z (PMC8481345; doi:10.1038/s41598-021-98651-z)
Supplement: Supplementary file 1 — Supplementary Information. [file 41598_2021_98651_MOESM1_ESM.pdf]

TNF induces glycolytic shift in Fibroblast like Synoviocytes via GLUT1 and HIF1A

Kathrin Koedderitzsch[1, 2], Ekaterina Zezina[1], Lingzi Li[1], Matthias Herrmann[1], Nadine Biesemann[1*]

[1]Sanofi R&D Immunology and Inflammation Therapeutic Area, Type 1/17 Inflammation and Arthritis Cluster, Industriepark Hoechst, 65926 Frankfurt am Main, Germany

[2]BioNTech, An der Goldgrube 12, 55131 Mainz, Germany

*corresponding author: Nadine Biesemann, nadine.biesemann@sanofi.com

Supplemental information

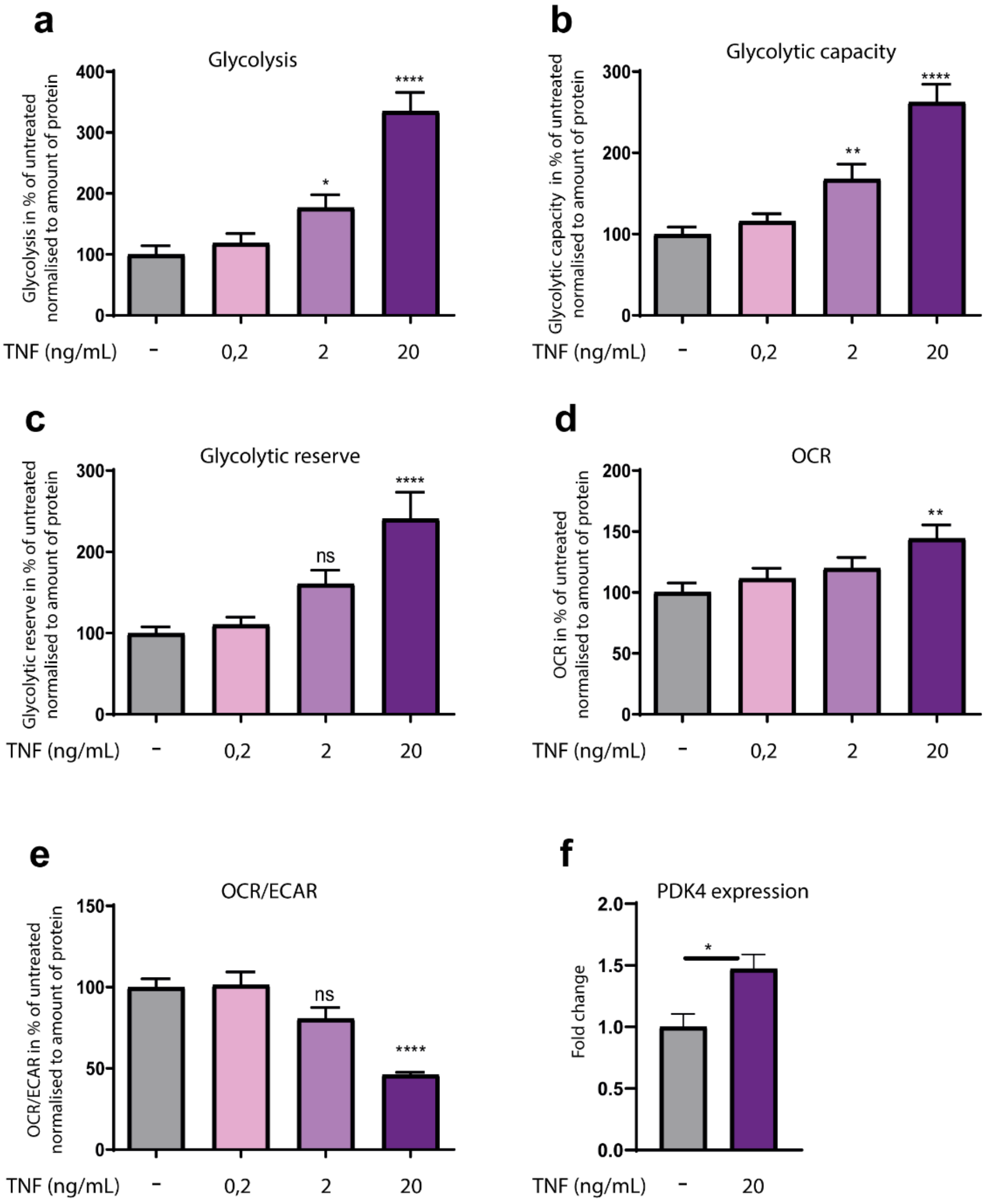

Suppl. Fig. 1 TNF also activates glycolysis and mitochondrial respiration in RA-FLS

(a-e) Real-time analysis of glycolytic function in RA-FLS from one donor after 24 h incubation with recombinant human TNF using Seahorse XFe96. Glycolysis stress test with injection of glucose (24 mM), oligomycin (1 µM) and 2-DG (100 mM). (a) Glycolysis, (b) Glycolytic capacity, (c) Glycolytic reserve, (d) OCR, (e) OCR/ECAR after glucose injection (experiments performed with 6 technical replicates, 2 independent experiments). Data were normalized to protein content using BCA assay. (f) PDK4 gene expression in RA-FLS. Fibroblast-like synoviocytes (one donor) were stimulated with recombinant human TNF (20 ng/ml) for 24h (experiments performed with 4 technical replicates, 2 independent experiments). Values represent means ±SEM. *P≤0.05; **P<0.01; ****P<0.0001, one-way Anova with Dunnett correction (a-e) or unpaired t-test (f).

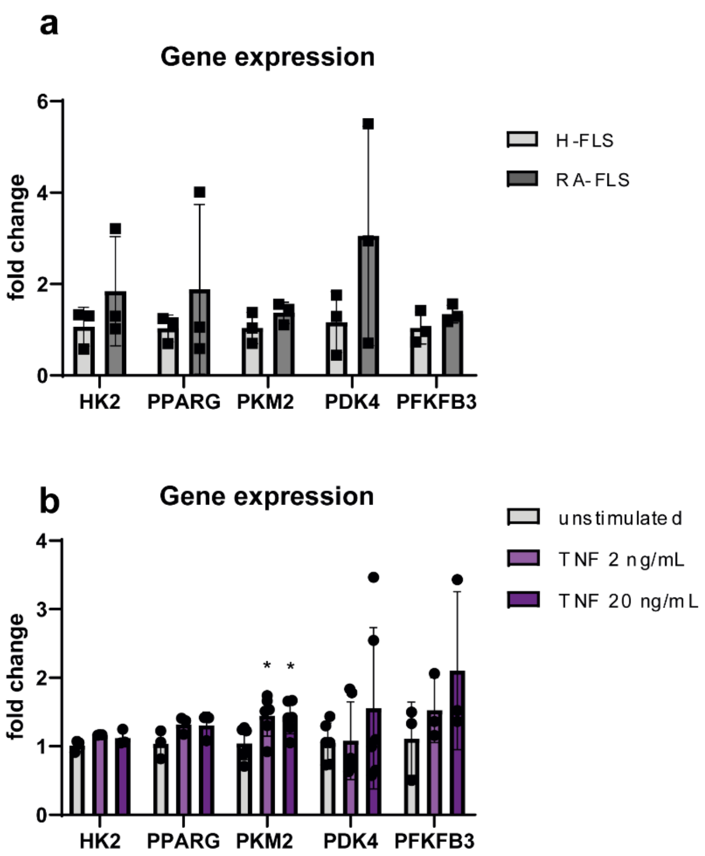

Suppl. Fig. 2 Upregulation of glycolytic genes in RA-FLS and H-FLS after TNF treatment

(a) Metabolic gene expression in fibroblast-like synoviocytes from healthy or RA donors (3 donors each). One dot represents one donor. (b) Metabolic gene expression in H-FLS after TNF stimulation. Fibroblast-like synoviocytes from three healthy donors were stimulated with recombinant human TNF (2 and 20 ng/ml) (1-2 independent experiments, n=3-6). Values represent means ±SEM, unpaired t-test (a), one-way Anova with Dunnett correction (b). *P≤0.05

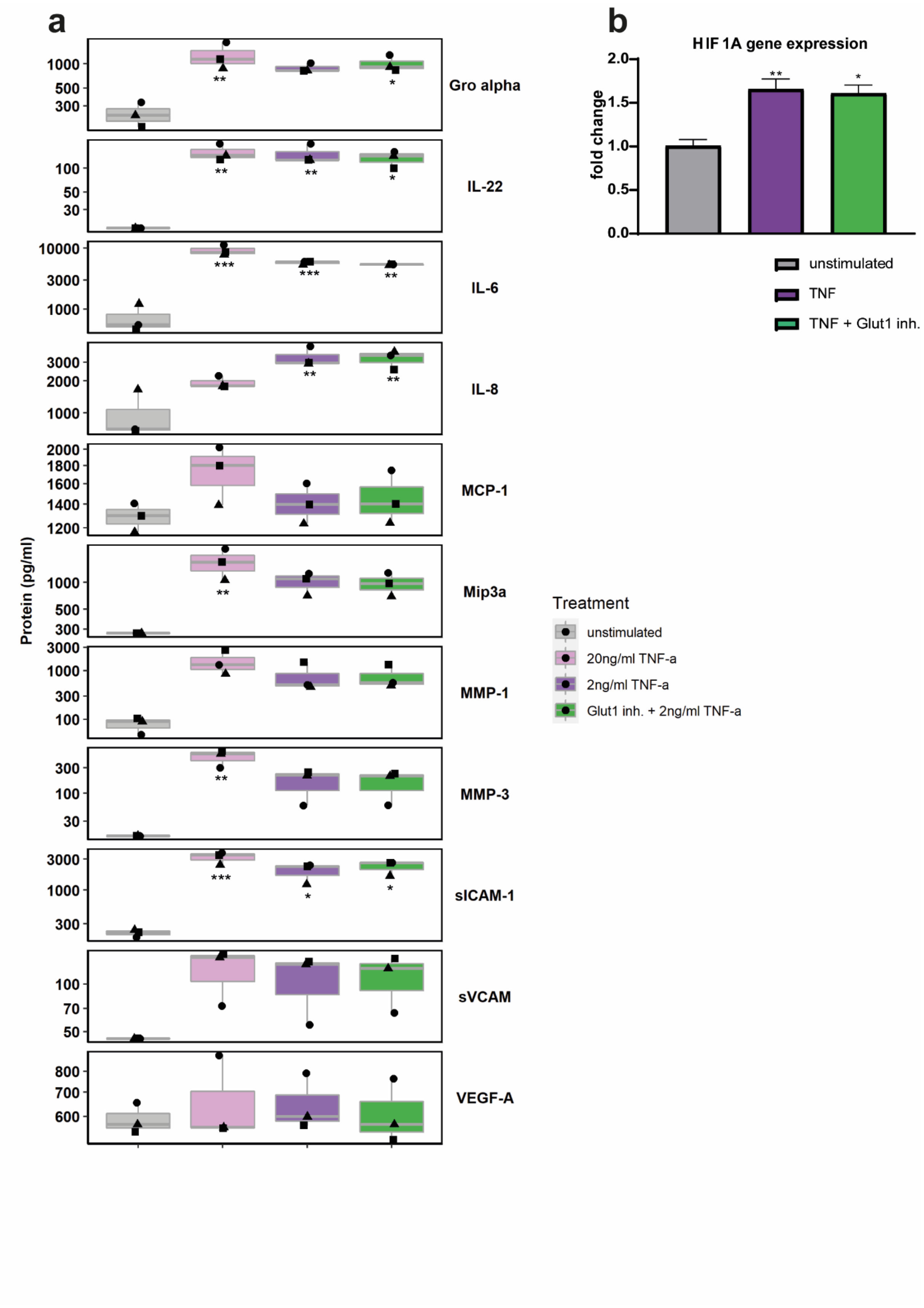

Suppl. Fig. 3 Glut1 is not essential for TNF's proinflammatory role

(a-b) H-FLS from three donors were stimulated with recombinant human TNF and a GLUT1 inhibitor (1 µM) for 24 h. (a) Luminex® Multiplex assay to evaluate cytokines and disease-relevant biomarkers in cell culture supernatants (3 donors, 1 experiment). One dot represents one donor. Data are shown as boxplot with median and first and third quartile. One-way ANOVA with Tukey multiple pairwise-comparisons. (b) HIF1A expression (n=3, 3 different donors). Values represent means ±SEM, unpaired t-test.

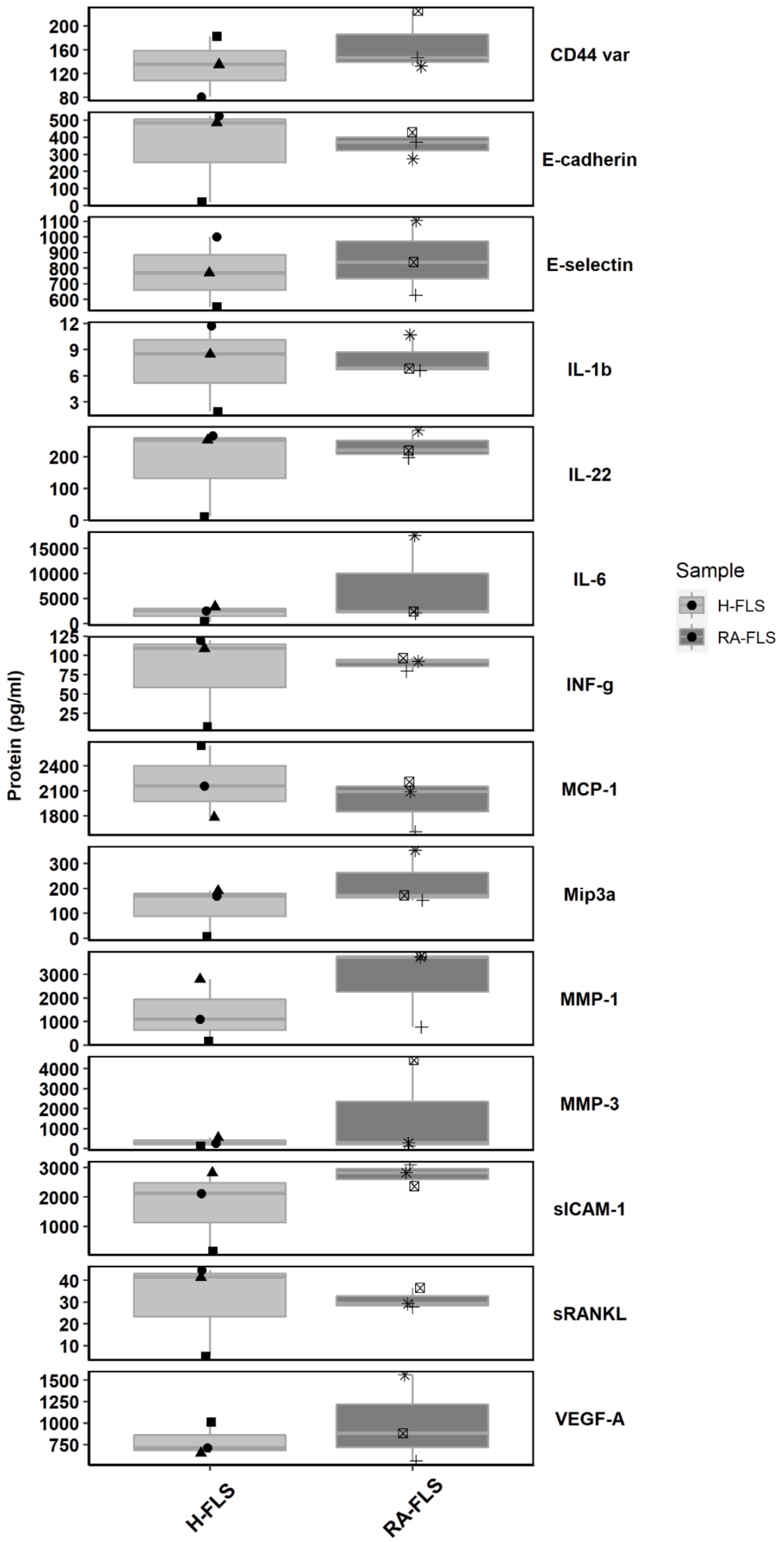

Suppl. Fig. 4 RA-FLS secrete slightly higher amounts of cytokines, chemokines and MMP's

Luminex® Multiplex assay to evaluate cytokines and disease-relevant biomarkers in cell culture supernatants (3 healthy and 3 RA donors). One dot represents one donor. Data are shown as boxplot with median and first and third quartile. Unpaired two-samples t-test.
